# Graft-versus-host disease: teaching old drugs new tricks at less cost

**DOI:** 10.3389/fimmu.2023.1225748

**Published:** 2023-08-03

**Authors:** Shatha Farhan, Shernan G. Holtan

**Affiliations:** ^1^ Stem Cell Transplant and Cellular Therapy, Henry Ford Health, Detroit, MI, United States; ^2^ Division of Hematology, Oncology, and Transplantation, University of Minnesota, Minneapolis, MN, United States

**Keywords:** graft-versus-host disease, drug repurposing, post-transplant cyclophosphamide, bortezomib, human chorionic gonadotropin, sitagliptin, a-1-antitrypsin

## Abstract

Graft-versus-host disease (GVHD) remains a major cause of morbidity and mortality after allogeneic stem cell transplantation (SCT). Currently, more patients can receive SCT. This is attributed to the use of reduced intensity regimens and the use of different GVHD prophylaxis that breaks the barrier of human leukocyte antigen, allowing an increase in the donor pool. Once an area with relatively few clinical trial options, there has been an increase in interest in GVHD prophylaxis and treatment, which has led to many US Food and Drug Administration (FDA) approvals. Although there is considerable excitement over novel therapies, many patients may not have access to them due to geographical or other resource constraints. In this review article, we summarize the latest evidence on how we can continue to repurpose drugs for GVHD prophylaxis and treatment. Drugs covered by our review include those that have been FDA approved for other uses for at least 15 years (since 2008); thus, they are likely to have generic equivalents available now or in the near future.

## 1 Introduction

Survival after allogeneic stem cell transplantation (SCT) has improved over the last few decades (1) and is related to many factors, including changes in condition regimens, infection prevention, and supportive care (2). Although graft-versus-host disease (GVHD) has been declining over the years (3) with lower rates of grade III/IV but stable grade II (4), it is still considered one of the major causes of morbidity and non-relapse mortality (NRM) (5).

Our understanding of GVHD development has expanded over the years beyond the interactions between host and donor cells (6) in the setting of tissue injury and cytokines after the conditioning regimen. Hence, GVHD prophylaxis and treatment have seen significant development and progress in recent years, which has led to many US Food and Drug Administration (FDA) approvals. However, some of these treatments are expensive, particularly if they need to be used by the patient for a long period of time. Despite having health insurance, patients with GVHD develop a financial strain that is associated with decreased quality of life (7). Repurposing (also known as repositioning) drugs with established safety profiles in humans may have the advantages of faster clinical trial times and lower costs (8). The ideal candidates for drug repurposing would be agents that are well tolerated, can be given for a finite period, and can prevent or treat GVHD while maintaining the graft versus leukemia (GVL) effect, without increasing the risk of infection, organ damage, or financial toxicity. The drugs covered by our review have been FDA approved and available for at least 15 years and include post-transplant cyclophosphamide (PTCy), abatacept, sitagliptin, α-1-antitrypsin (AAT), vitamin A, bortezomib, human chorionic gonadotropin (hCG), and lithium. The list is not meant to be comprehensive.

## 2 Examples of drugs applied in general practice

### 2.1 Post-transplant cyclophosphamide

The most important development in the prevention of both acute GVHD (aGVHD) and chronic GVHD (cGVHD) over the past few decades is PTCy. There has been an increase in the use of PTCy in different donor types after its successful use in haploidentical SCT (9). In the 1960s, Berenbaum and Brown reported that the use of single-dose cyclophosphamide in mice was effective in prolonging skin homo-graft survival if administered any time from shortly after grafting to day +4, but not if used before grafting or on day +6 (10). Luznik et al. were able to show that PTCy decreases the incidence and severity of aGVHD after transplantation of major histocompatibility complex-incompatible marrow in mice given cyclophosphamide 200 mg/kg intraperitoneally on day 3 (11). Luznik and his team also showed that in the haploidentical bone marrow SCT setting, there was a trend toward less extensive cGVHD in patients who received cyclophosphamide 50 mg/kg intravenously on days 3 and 4 versus only day 3 after SCT (12). This started a great change in the field of GVHD prevention, although the exact mechanism of action, best dose, and effect on immune reconstitution post-SCT are still not completely understood. It has been postulated that PTCy might decrease alloreactive T cells but not completely eliminate them. Alloreactive regulatory T cells (Tregs) (13) are resistant to PTCy due to high aldehyde dehydrogenase expression post-SCT. When Kanakry and his team examined CD4+CD25−Foxp3− donor T cells in murine models, they found that although at day +7 these cells were lower in number than the control, they remained within the same log range, and their expansion between day +7 and day +21 was constrained using PTCy. It is thought that the preferential *in vivo* expansion of the Tregs in the first three weeks post-SCT has contributed to the restrained expansion of conventional T cells because depletion of Foxp3+ resulted in GVHD (14). PTCy was also peculiar compared to five other chemotherapies (methotrexate [MTX], bendamustine, paclitaxel, vincristine, and cytarabine) in this property of constraining alloreactive T cells on days +7 and +21 while reconstituting Tregs at day 21 (15). In mouse models, Fletcher et al. showed that this PTCy effect on Tregs is indirect by modifying the immune environment and expansion of myeloid-derived suppressor cells (16). In addition, PTCy has a possible effect on natural killer (NK) cells (17–19), the early recovery of which is important for better overall survival (OS) post-SCT (18) due to decreases in both relapse and NRM. Studies are examining 50%, 30%, or 20% reductions in the dose, with or without the addition of other agents, with preliminary data showing faster engraftment and less toxicity; however, longer follow-up is needed to determine the effect on cGVHD (20–22).

After the success in the haploidentical setting, many retrospective studies have examined PTCy use in mismatched unrelated donors (MMUDs) (23–25). A prospective study evaluating PTCy in MMUD peripheral blood (PB) SCT for patients with hematological malignancies was reported by Al Malki et al. (26). Myeloablative conditioning (MAC) and reduced intensity conditioning (RIC) regimens were used. After 18 months of follow-up, 18% of patients had grade III–IV aGVHD at 100 days. Moderate-to-severe cGVHD at 1 year was 3%, with GVHD-free, relapse-free survival (GRFS) of 68% at 1 year. BK virus cystitis was seen in five patients, mostly grade I. Cytokine release syndrome was seen more frequently in the RIC arm than in the MAC arm. Another study, sponsored by the National Marrow Donor Program, by Shaw et al. showed the success of bone marrow (BM) with PTCy and sirolimus in the MMUD setting (27). This prospective phase II study included 80 patients. Both MAC and RIC regimens were used. Forty-eight percent of patients enrolled were of ethnic minorities: 19% were African American, and 24% were Hispanic or Latino; 39% of patients received four to six out of eight HLA MMUDs. The study showed that almost 18% of patients who received MAC developed aGVHD grade III–IV at day 100, with no aGVHD grade III–IV at day 100 in the RIC group. GRFS and NRM at 1 year were 38% and 8%, respectively, in patients receiving MAC compared to 55% and 10%, respectively, in patients receiving RIC. The updated report showed a 3-year OS and NRM of 70% and 15% in the RIC group and 62% and 10% in the MAC group, respectively. However, there was a high rate of relapse especially in the MAC group, reaching 51% at 3 years, although the high-risk disease index was 8% and intermediate risk was 73%. All patients received BM grafts (28). Currently, the National Marrow Donor Program study ACCESS is open for accrual for PB MMUDs with PTCy, tacrolimus, and mycophenolate mofetil (MMF) (NCT04904588).

In matched donors, a prospective randomized phase II study by Brissot et al. (29) comparing antithymocyte globulin (ATG) versus PTCy in matched related donors (MRDs) and matched unrelated donors (MUDs) PB RIC SCT showed no significant difference between the two in terms of GRFS, relapse, or NRM. All 80 patients received a conditioning regimen of fludarabine and busulfan and were randomized to receive PTCy with cyclosporin A (CsA) from day +5 or ATG 2.5 mg/kg per day on days −2 and −1 with CsA from day −3. At 6 months, the cumulative incidence (CI) of grade II–IV GVHD was 34.9% in PTCy versus 24.3% in the ATG arm (p = 0.53), and grade III–IV was 9.3% versus 2.7% (p = 0.24). The 1-year CI of cGVHD was 26.0 in PTCY versus 30.2 in ATG recipients (p = 0.56). The 1-year estimated OS and GRFS were 78.9% and 52.2%, respectively, in the PTCy group and 80.4% and 42.2%, respectively, in the ATG group; none were statistically significant. In addition, in the setting of RIC PB SCT in MRDs and MUDs, a phase III randomized trial comparing tacrolimus/MTX to PTCy-based GVHD prophylaxis (Blood and Marrow Transplant Clinical Trials Network [BMT CTN] 1703 NCT03959241) was recently reported at the 64^th^ ASH Annual Meeting (30). This study randomized 431 patients and showed a significant difference in the adjusted 1-year GRFS rate between the two arms. GRFS in the PTCy arm was 52.7% versus 34.9% for the control arm. Most patients had MUDs or MRDs except for 3.5% who are MMUDs. The day 100 grade III–IV aGVHD was 6.3% versus 14.7% (p = 0.001), and the cGVHD rate at 1 year was 21.9% versus 35.1% (p = 0.005) for PTCy versus tacrolimus/MTX, respectively. There was no difference in the relapse/progression or OS rate at 1 year. The cumulative incidence of engraftment was lower for PTCy for neutrophils ≥ 500/mm^3^ by day +28 (90.3% versus 93.4%, p = 0.03), platelets ≥ 50,000/mm^3^ by day +100 (79.5% versus 83.7%, p < 0.001), and lymphocytes ≥ 1,000/mm^3^ by 1 year (47.1% versus 63.2%, p < 0.001). In the MRD or MUD PB but with non-myeloablative setting, the HOVON-96 trial (31) examined PTCy/CsA versus a combination of CsA and MMF. The CI of II–IV aGVHD at 6 months was 48% in recipients of CsA and MMF versus 30% following PTCy/CsA (p = 0.007) with a 1-year estimate of GRFS of 21% versus 45%, p < 0.001, respectively.

Increased risk of infection is a concern with the use of PTCy, especially when used at the current dose of 50 mg/kg on days +3 and +4. This could be due to the *in vivo* lymphodepletion and delayed immune reconstitution caused by PTCy itself or could be related to the degree of mismatch or both. Camargo et al. (32) assessed the incidence of any cytomegalovirus (CMV) viremia and clinically significant viremia, which they defined as CMV disease or CMV viremia leading to preemptive treatment. They compared the results between PTCy MMUDs, ATG MMUDs, and PTCy haploidentical transplants. Ninety percent of the patients in the PTCy MMUDs had a BM transplant. The rate of clinically significant CMV viremia was lower in PTCy MMUDs compared to PTCy haploidentical and ATG MMUDs and remained significantly lower after adjusting for letermovir prophylaxis. A similar report by Irene et al. (33), who examined all patients who received PTCy as GVHD prophylaxis regardless of donor type, found that CMV infection and viral hemorrhagic cystitis were higher in the haploidentical SCT cohort compared to MRDs or MUDs/MMUDs (58% versus 43% and 30% versus 8% on day +90, p < 0.05). However, in a large Center for International Blood and Marrow Transplantation Research (CIBMTR) study (34), PTCy increased the risk of CMV infection in both MRD and haploidentical SCT using PTCy. In another CIBMTR study (35) comparing haploidentical versus MUDs both with PTCY as GVHD prophylaxis, the NRM was higher in the haploidentical cohort in the RIC group. However, they did not observe differences in viral infections, while data on other infections were available for only a small subset of patients. In the prospective setting, in the 1703 study (30), CMV reactivation and grade 3 infection rates were similar between the arms, but grade 2 infections were greater in the PTCy/tacrolimus/MMF group compared to tacrolimus/MTX (33.7% versus 23.5%, p = 0.002). In addition, there was more organ failure as the cause of death in the PTCy/tacrolimus/MMF approximately 23% versus 11% in the tacrolimus/MTX (36). In the Brissot et al. study, CMV reactivation was similar between the two groups. Although numerically Epstein–Barr virus was slightly higher in the ATG group, and cardiac and hemorrhagic cystitis were higher in the PTCy group, none reached statistical significance. Also, in the HOVON study (31), Common Terminology Criteria for Adverse Events grade 3 to 5 infections were observed in 21% versus 41% CsA/MMF versus PTCy/CsA, with similar rates of CMV reactivation. Regarding grade 3–5 adverse events within 6 months post-SCT, the percentage was 42% in the CsA/MMF arm versus 61% in the PTCy/CsA arm. Organ failure as the cause of death was reported in 6% of the CsA/MMF arm versus 3% in the PTCy/CsA arm.

Although PTCy might cost less than other GVHD prophylaxis (37) (**Table 1**), the potential need for a longer hospital stay, slower engraftment (53), and T-cell immune reconstitution, which can lead to infections and BK cystitis, might potentially add more to the cost. However, the significant reduction in aGVHD and cGVHD with fewer patients needing immune suppression (54, 55) may decrease the overall cost and health care burden. Yu et al. (56) found that patients with aGVHD, including those with steroid-refractory or high-risk aGVHD, had longer median lengths of stay and higher median total costs when compared with patients with no GVHD ($153,849 and $205,880 versus $97,417). More studies are needed to examine the cost-effectiveness of PTCy versus other GVHD prophylaxis.

**Table 1:** Selected drugs that are being repurposed for prevention or treatment of graft-versus-host disease.

Drug	Price^*^	Ref	Prevention aGVHD	Prevention cGVHD	Treatment aGVHD	Treatment cGVHD	Year of FDA approval for other indication
**Post-transplant cyclophosphamide**	2 g/10 mL intravenous solution (per mL): $175.80 (38)	(30)	Yes	Yes	No	No	1999
**Abatacept**	250 mg intravenous solution: $1,617.07 (39)	(40)	Yes	No	No	CT	2005
**Sitagliptin**	100 mg per tablet: $21.89 (41)	(42)	Yes	No	No	No	2006
**Alpha-1-antitrypsin**	1,000 mg/50 mL solution: $0.74 (43)	(44)	CT	CT	No	No	1987
**Vitamin A**	3 mg (10,000 UT) per capsule: $0.02–$0.05 (45)	(46)	Yes	Yes	No	No	FDA does not approve dietary supplements but plays a role in regulating them
**Bortezomib**	2.5 mg injectable powder: $230.41 (47)	(48)	CT	CT	No	No	2003
**Human chorionic gonadotropin**	10,000 units per vial: $139.51 (49)	(50)	CT	CT	Yes	CT	1976
**Lithium**	450 mg per controlled-release tablet: $0.46–$0.76 (51)	(52)	CT	CT	CT	CT	1970

CT, clinical trials; FDA, Food and Drug Administration; GVHD, graft-versus-host disease; Ref, reference.

^*^Sources of the pricing information are cited within each cell.

### 2.2 Antithymocyte globulin

ATG interferes with the immune response with multiple proposed mechanisms of action that may include depletion of T cells (57), apoptosis of B cells (58), and effect on dendritic cells (59). ATG has been studied extensively over the years as GVHD prophylaxis in the setting of MRD, MUD, MMUD, and haploidentical SCT but with differences in terms of patient populations, donors, stem cell source, regimens, timing, dose, formulations, relation to absolute lymphocyte count (60), and planned use of granulocyte colony-stimulating factor (GCSF) (61). Two recent meta-analyses by Kumar et al. (62) and Yang et al. (63) examined ATG in SCT GVHD prevention. Both meta-analyses suggested that ATG reduced grade II/III and grade III/IV aGVHD and cGVHD without affecting the OS and NRM. However, there was a difference in the risk of relapse, with the former suggesting an increase in relapse that was not seen in the latter, probably due to studies that included longer follow-up periods.

Admiraal et al. reported the results of a prospective, single-arm, phase 2 clinical trial investigating a new way of using an old drug by applying individualized dosing of ATG for unrelated SCT in pediatrics (64). They based the dosing on body weight, absolute lymphocyte counts before the first dose, and the stem-cell source, with cumulative doses ranging from 2 to 10 mg/kg. This dosing method improved early CD4+ immune reconstitution (80% of evaluable patients) without increasing GVHD, relapse, or graft failure.

### 2.3 Abatacept

Abatacept is a recombinant soluble fusion protein that targets T-cell costimulation by binding to CD80/86 more avidly than CD28 (65). It was approved by the FDA for the treatment of rheumatoid arthritis in 2005. Recently, there has been more interest in targeting the many T cells’ costimulatory pathways including CD28/CTLA4:CD80/86, OX40 (CD134):OX40L (CD252) (66), and CD40L:CD40 (67). This interest was not only in autoimmune diseases but also in transplantation, like using abatacept in the prevention of GVHD in SCT and using belatacept, a second-generation CTLA4-Ig, to prevent graft rejection in solid organ transplants (68). Multiple studies in murine and primate models examined the effect of disrupting the CD28/CTLA4:CD80/86 pathway and found a decrease in GVHD (69–71). Clinically, the addition of abatacept to GVHD prophylaxis was shown to reduce the incidence of severe aGVHD after MUD and MMUD SCT. It was first tested in a small feasibility study that showed it was safe before moving forward to larger studies. ABA1 (72) was a single-arm study that involved patients at high risk of developing aGVHD who received abatacept added to the standard of care: calcineurin inhibitors and MTX. Abatacept was administered in four doses on days −1, +5, +14, and +28. This was followed by ABA2 (phase II clinical trial) (40). For patients receiving MUD SCT, a randomized double-blind placebo-controlled design was used, with patients randomly assigned to abatacept or placebo. While for the 7/8 MMUDs, due to low recruitment, likely due to them being at high risk of severe GVHD, the trial was amended such that all patients receiving 7/8 MMUDs were assigned to calcineurin inhibition and MTX plus abatacept as an open-label single-arm stratum. Of note, this study included a significant number of pediatric patients, with MAC in almost 2/3 of the patients plus significant use of BM at almost 50%. In MUD transplants, there was a significant benefit regarding grade III–IV aGVHD; the rate was 6.8% in the abatacept group versus 14.8% in the placebo group. In the smaller 7/8 cohort, there was also a notable difference in rates of developing grade III–IV aGVHD; the rate was 2.3%, which compared favorably with a retrospective CIBMTR cohort of CNI/MTX (30.2%), treated without ATG or PTCy. In addition, a *post-hoc* analysis was performed to compare the 7/8 ABA group to a retrospective CIBMTR 7/8 cohort who received CNI/MTX+ATG. In this analysis, grade II–IV aGVHD was the same, 40% versus 42%, respectively; however, there was a reduction in grade III–IV aGVHD, 3% versus 22%, respectively. In both the 7/8 and 8/8 cohorts, day +180 severe aGVHD free-survival outcomes for patients receiving abatacept were superior to those receiving standard prophylaxis, and hence, the FDA approved the drug for aGVHD prophylaxis in unrelated donors in 2021. There was no statistically significant difference in CMV or Epstein–Barr virus viral reactivation or end-organ disease in 8/8 patients, a finding that helped confirm the safety of adding abatacept to the GVHD prophylactic regimen. Viral reactivation data were not collected by the CIBMTR, so 7/8 patients could not be directly compared for this endpoint.

ABA1 and ABA2 studied four doses of abatacept, and both did not show an effect on cGVHD development (40), an outcome that might be expected given the limited exposure to abatacept, with the last dose being at day +28. Smaller studies have suggested that abatacept may have activity in treating cGVHD (73–75) with one showing in its correlative studies a reduction in interleukin (IL)-1-alpha, IL-21, and tumor necrosis factor (TNF)-alpha post-abatacept (75). These cytokines may play a role in B-cell modulation through T follicular helper cells. Therefore, increasing the number of abatacept doses could prevent both cGVHD and aGVHD. Thus, there is a rationale for testing an extended dosing schedule (eight doses versus four doses) to determine whether longer exposure could improve cGVHD outcomes in ABA3 (NCT04380740). Another way to try to decrease cGVHD using abatacept is to combine it with PTCy. There are ongoing studies examining combining PTCy and abatacept for GVHD prophylaxis. Some of the studies that examined this combination are also about non-malignant disorders, like aplastic anemia (76) and hemoglobinopathies (77). In the study by Jaiswal et al. (77), there were no cases of aGVHD or cGVHD, and all nine patients were off sirolimus as planned after day +270. The CAST trial (NCT04503616) examines a combination of PTCy, abatacept, and a short course of tacrolimus for GVHD prevention following haploidentical PB SCT; the regimen consists of PTCy on days +3 and +4 and abatacept on days +5, +14 and +28, and tacrolimus with taper was initiated on day +60 and completed by day +90. Results were updated at the 64th ASH Annual Meeting (78) and recently published (79); of the 46 enrolled patients, 41.3% were from racial or ethnic minorities. Day +120 CI of aGVHD grades II–IV, III–IV, and IV with death as a competing event was 17.4%, 4.4%, and 0%, respectively. One-year CI of moderate-to-severe cGVHD was 15.9%. A different phase II randomized clinical trial (NCT03680092) is comparing GVHD prophylaxis with PTCy and abatacept (CNI-free regimen) with CNI/mini dose MTX in 8/8 MRDs or MUDs. Preliminary results were presented at the 63^rd^ ASH Annual Meeting in 2021 (80). In this trial, patients in the PTCy arm received abatacept on days +5, +14, +28, +56, +84, +112, +140, and +168. The primary endpoint is cGVHD at 1 year. Ten of the 25 patients enrolled were in the PTCy/abatacept arm, and none had grade III–IV aGVHD or cGVHD after a follow-up of 516 days. None had engraftment failure or NRM. These active clinical trials in MUD/MRD and haploidentical SCT mainly show that the combination of PTCy and abatacept is feasible and provide the foundation for more trials to assess the use of abatacept in MMUDs. One of them is the ongoing phase I–II clinical trial for SCT from an MRD or ≥7 out of 8 unrelated donors. Subjects will receive PTCy, bortezomib, and abatacept as GVHD prophylaxis (NCT05289167).

Regarding relapse in the ABA2 study, there was no apparent increase in relapse in ABA patients. Abatacept was also compared retrospectively to two cohorts of patients in the CIBMTR registry, one with ATG and one with PTCy-based GVHD prevention in 7/8 MMUDs and 8/8 MUDs, respectively. Abatacept had a statistically significant better 1-year OS and relapse-free survival when compared to ATG. However, when compared to the PTCy cohort, there was a trend to better OS and relapse-free survival in the abatacept group, but it was not statistically significant (81). This observation of less relapse in the ABA group could possibly be related to the early transient inversion in the percentage of T cells versus NK cells in favor of NK cells approximately day +28 post-SCT (72). Alternatively, the lack of cGVHD mitigation with four doses could also explain the better relapse-free survival in the ABA group since some studies showed that patients with cGVHD might have a decreased risk of relapse (82).

## 3 Examples of drugs that are being used within the context of clinical trials or in development for clinical trials

### 3.1 Sitagliptin

Sitagliptin is one of many dipeptidyl peptidase-4 (DPP4) inhibitor groups of medications that have been used over many years to control type 2 diabetes mellitus (83) and approved by the FDA in 2006. By inhibiting DPP4, the half-life of glucagon-like peptides (GLPs) is increased, which in turn enhances endogenous insulin secretion (84). Interestingly, the lymphocyte cell surface protein CD26 possesses DPP4 activity (85). In addition, the CD26/DPP4 homodimer is expressed in many tissues like the lung and intestine and many other immune cells like B and T cells, activated NK cells, and myeloid cells (86, 87). The relationship between CD26/DPP4 and T-cell simulation is very complex but involves direct and indirect pathways. In terms of cytokines, *in vitro* studies have shown that DPP4 inhibition resulted in a decrease in activating cytokines like IL-2 and IL-6 but an increase in the secretion of transforming growth factor-beta-1 (88). In addition, CD26/DPP4 interacts directly with antigen-presenting cells via caveolin-1, resulting in the upregulation of costimulatory molecule CD86 and hence triggering T-cell activation and proliferation via the nuclear factor-kappa B pathway (89).

Farag et al. were interested in studying DPP4 inhibitors in improving engraftment post-umbilical cord blood SCT. Interestingly, in the course of conducting their studies, they observed that patients were developing GVHD at a lower rate than the historical data. This observation led to a formal study of sitagliptin at a dose of 1,200 mg/day in addition to tacrolimus and sirolimus in patients with MRDs or unrelated donors receiving MAC regimens in a phase II non-randomized clinical trial. Sitagliptin was given for 2 weeks starting the day of the transplant, while tacrolimus and sirolimus were tapered at day 100 and discontinued by approximately day 180. In the event of renal toxic effects, MMF (1,000 mg twice daily) was substituted for tacrolimus at the physicians’ discretion. By day 100, only two of 36 patients developed aGVHD, with rates of aGVHD II–IV of 5% and III–IV of 3%. Relapse and NRM were 26% and zero at 1 year, respectively. CI of cGVHD at 1 year was 37%, with seven of the 15 patients who developed cGVHD having moderate cGVHD and three with severe cGVHD. One-year GRFS was 46% (42). During the trial, since this is an oral medication given to patients who had MAC regimens that cause significant mucositis, nausea, and vomiting, only 28 patients received 80% or more of the planned 32 doses of sitagliptin, and one patient could not receive the planned dose because of acute renal failure since sitagliptin is mainly excreted in the urine and that patient had grade IV aGVHD by day 100. The two patients who had grades II and IV aGVHD by day 100 received 65% and 70% of the planned total dose, respectively, and both received PB stem cells from unrelated donors.

Interestingly, the effect of sitagliptin on gut microbiome was examined by investigators in mouse models (90) and in adults with diabetes mellitus (91) with a possible effect in the mouse models but no effect on microbiome diversity in adult patients with diabetes mellitus. However, the dose used for diabetes mellitus is 12 times lower than what was used by Farag et al. In addition, there is a recent increase in interest in the effect of GLPs in maintaining homeostasis in the gut, as they are produced by intestinal L cells. GLPs are rapidly degraded by DPP4, and their inhibition has gained attention in inflammatory bowel disease research (92). This approach will need further evaluation in randomized clinical trials, possibly best in a RIC setting with a combination of other drugs like low-dose PTCy with a lower risk of severe mucositis and renal failure to be able to take the full course.

Another way to increase the half-life of GLPs including GLP2, an enteroendocrine hormone, is the development of the degradation-resistant GLP2 analogue termed teduglutide. This drug was approved by the FDA in 2012 for short bowel syndrome. Norona et al. (93) showed in a mouse model that GVHD depletes GLP2+ L cells. Thus, they examined the effect of treating mice with teduglutide from day −3 to +3 post-SCT and found that there is a decrease in GVHD-related death and a decrease in GVHD histopathology scores in these mice. In mice treated from day −3 to +10, teduglutide reduced GVHD-induced Paneth cell loss, modulated intestinal microbiome, and promoted intestinal stem cells without loss of GVL. In patients, when the blood concentration of GLP2 at the time of diagnosis of GVHD was examined, higher levels were associated with a higher incidence of steroid-refractory (SR) GVHD and NRM. Zeiser and colleagues opened the trial NCT04290429 to use this drug in the treatment of patients with SR gastrointestinal (GI) GVHD, and another trial is NCT05415410, examining the use of apraglutide, a similar medication with a potentially better safety profile and less frequent subcutaneous administration.

### 3.2 Alpha-1-antitrypsin

AAT is a plasma glycoprotein that is produced by hepatocytes. It has a unique three-dimensional structure (94) that allows it to have many hydrophobic and electrostatic interactions with other proteins and peptides, mediating an anti-inflammatory effect. It was originally approved by the FDA in 1987 to treat emphysema associated with AAT deficiency. This led to many products in the market, most of which are derived from pooled human plasma. There are a growing number of preclinical and clinical trials studying the effect of AAT and its potential use in autoimmune diseases, including lupus (95).

Marcondes et al. (96) showed retrospectively the inverse correlation between AAT plasma level in MRDs and the risk of aGVHD in 111 patients. Since the amount of donor plasma transferred is minimal and does not explain this, they tested the hypothesis that AAT-exposed donor cells, not the AAT level itself, is the cause of this observation in murine transplant models. Mice given cells from AAT-treated donors had less weight loss, lower GVHD incidence and severity, and reduced mortality when compared with albumin controls. This was the result of an increase in Treg expansion that was dendritic cell-dependent, enhanced anti-inflammatory cytokines like IL-10, and decreased proinflammatory cytokines like TNF-alpha and IL-1b. Furthermore, there was an increase in NK cells with potent antitumor cytotoxicity preserving the GVL effect. In their models, the effect of treating the donor with AAT was comparable or superior to that of direct treatment of the recipients in GVHD prevention, and the benefit was further enhanced when both donor and recipient were treated. In the 1980s, Weisdorf et al. (97) measured fecal AAT in 25 SCT patients and found that patients who developed GI aGVHD had an increase in AAT loss in stool. Based on these results, Marcondes et al. (98) conducted a dose escalation study in SR GVHD with GI involvement where AAT was infused every other day for 2 weeks, and they found no clinically apparent toxicity in any patient. However, there was a flare of aGVHD after the completion of therapy, and some patients required treatment for cGVHD. In a phase II trial, Magenau et al. (44) extended the treatment to 4 weeks by giving 40 patients with SR aGVHD AAT twice weekly at a dose of 60 mg/kg per day for up to 4 consecutive weeks (maximum eight doses). The treatment was well-tolerated and resulted in an overall response of 65% by day 28. Based on the above, there are ongoing prospective studies examining the role of AAT in the prevention or treatment of GVHD including MODULAATE (NCT03805789) and BMT CTN 1705 (NCT04167514).

### 3.3 Vitamin A

Vitamin A is involved in many biological processes including the regulation of immune responses via targeting T cells, B cells, antigen-presenting cells, and innate lymphoid cells, with most of its effect being related to its major metabolite retinoic acid (RA) (99). RA signaling is mediated by heterodimers of RA receptors and retinoic X receptors (RXRs) (100). Interestingly, RA not only augments cell migration to the intestines by inducing the expression of molecules like CCR9 (101) but also plays a role in modulating intestinal CD4+ T cells, enhancing the stability of natural Tregs and the conversion of naïve T cells into induced Tregs (102).

The team at Cincinnati Children’s Hospital reported increased gut GVHD in children with lower levels of vitamin A measured in plasma at day 30 post-SCT (46). They recently presented their prospective randomized double-blinded, placebo-controlled clinical trial where patients with vitamin A <75^th^ percentile of normal for age were randomized to receive high-dose oral vitamin A or placebo pre-SCT (103). In their “as treated” analysis, acute grade II–IV GVHD in placebo versus vitamin A arm was 10% versus 0%, respectively, with p = 0.049. GI aGVHD was higher in the placebo versus vitamin A groups (10% versus 0%; p = 0.049). cGVHD was also increased in placebo versus vitamin A (15.3% versus 2.7%, p = 0.009). Asymptomatic grade 3 hyperbilirubinemia possibly attributable to therapy was observed in one vitamin A patient, which self-resolved. They also examined CCR9+ CD8+ effector memory T cells at day 30 reflecting gut trafficking and CD4+CD25+CD127+ regulatory T cells at day +100; the former was higher, and the latter was lower in the placebo group. It is important to mention that they administered vitamin A as a single oral dose of 4,000 IU/kg prior to conditioning, before the start of the inflammatory environment that occurs later in the SCT process where RA can play an important role in CD4+ T-cell activation (104) and effector function since RA can have complex pro- or anti-inflammatory functions depending on the context (105).

Along the same lines and effect of the RXR axis in GVHD prevention while maintaining the GVL effect, Thangavelu et al. (106) reported the effect of IRX4204, which is a novel RXR agonist that activates RXR homodimers but not heterodimers. They reported reduced intestinal injury and promotion of Tregs in transplanted mice that received IRX4204 intraperitoneally from day 0 to day +56 post-SCT. The investigators showed that IRX4204 promotes and maintains Tregs by converting the donor FoxP3 T cells into sustained FoxP3+.

### 3.4 Bortezomib

Bortezomib is a proteasome inhibitor that is approved for the treatment of myeloma and mantle cell lymphoma and was found to have many biological effects including its anti-apoptotic effect mediated by blocking the activation of nuclear factor-kappa B pathway (107). This nuclear factor-kappa B and proteasome pathway was also found to play a role in cytokine signaling and immune response (108) and T-cell activation and proliferation (109). Interestingly, both Wang et al. (110) and Sun et al. (111) reported that the inhibitory effect of bortezomib on dendritic cells and alloreactive T cells, respectively, was dose- and time-dependent. Wang et al. also showed that it blocks the expression of co-receptors CD80 and CD86 and secretion of cytokines IL-12 (110) and TNF-alpha (110, 111) and, hence, constrains the ability of dendritic cells to activate T cells. Sun et al. examined not only the effect of early administration of bortezomib (days 0–3) in GVHD prevention but also the effect on maintaining GVL in tumor-bearing mice. They found that GVL was promoted only when bone marrow transplants, splenic cells, and bortezomib were administered, which can point to the indirect immune-mediated antitumor effect of bortezomib. However, when bortezomib is given at a later time post-transplant (days 5–7) or given early and then continued with the delayed administration, there was increased GVHD-dependent morbidity and gut toxicity (112).

Based on the promising results of a phase I/II trial of administering a short course of bortezomib in 45 patients with MMUD SCT with a day +180 rate of grade II–IV aGVHD of 22% (113), bortezomib was one of the arms of the BMT CTN 1203 prospective study. This study compared GVHD prophylaxis with either PTCy/tacrolimus/MMF or tacrolimus/MTX plus bortezomib or maraviroc with contemporary tacrolimus/MTX controls. The hazard ratio for GRFS was 0.72 in the PTCy arm compared to controls and with better performance than bortezomib and maraviroc. The most common toxicities were hematological toxicity and cardiotoxicity in all three arms (114). Previously published by Al-Homsi et al. (115) and updated at the 63^rd^ ASH Annual Meeting in 2021 (48), there was a study in patients receiving MUD transplantation who also received r-ATG and PTCy with two doses of bortezomib given 6 h after graft infusion and 72 h thereafter where all GVHD prophylaxis was completed on day +4. The rates of aGVHD grades II–IV and III–IV were 35.9% and 11.7%, respectively. The rate of cGVHD was 27%. The 2-year GRFS was 37.7%.

We recently presented the update of our experience at the 48^th^ Annual Meeting of the European Society for Blood and Marrow Transplantation (116) of a pilot study incorporating the addition of bortezomib to PTCy without ATG in the setting of PB haploidentical SCT. Seven patients were enrolled so far. Five patients had leukemia, and two had concomitant acute lymphoblastic leukemia and myeloma. Four patients were African American, two were White, and one was Hispanic. Chimerism post-SCT was ≥99% donor at day +30 for all patients. All six of the alive patients are off tacrolimus, with a median time to discontinuation of 203 days (186–218). Six patients had aGVHD with aGVHD II–IV of 28% and aGVHD III–IV of 14%; none had grade IV. None of the patients are currently on steroids or any immune suppression. Of the seven patients who were evaluable, one developed moderate cGVHD and is now off immune suppression. More research is needed to explore the effect of bortezomib in GVHD and GVL by examining the optimum dose, timing, and combination with other T-cell modifiers or the number of T cells infused.

### 3.5 Human chorionic gonadotropin

Although the mechanism of immune tolerance and the observation of improvement in autoimmune diseases, such as Crohn’s disease (117) and multiple sclerosis (118), during pregnancy is poorly understood, there are a few observations regarding the possible role of hCG. Since dendritic cells are critical in immunity and tolerance, Dauven et al. examined the effect of hCG on bone marrow-derived dendritic cells *in vitro*, which were significantly reduced in number when compared to those not treated with hCG (119). An intracellular enzyme known as indoleamine 2,3-dioxygenase (IDO) is important in the tryptophan degradation pathway, which in turn has immunomodulatory effects including suppression of T-cell proliferation (120). Jasperson et al. found that IDO^−/−^ mice developed very severe aGVHD post-transplant (121). IDO can be induced by inflammatory cytokines, but it was also found by Koldehoff et al. (122) to be increased in women treated with hCG as part of their *in vitro* fertilization induction. They found that hCG treatment resulted in a more pronounced response toward T helper 2 differentiation and an increase of Tregs. In addition, they reported that the rejection of allogeneic skin grafts in female mice was significantly delayed by using hCG (122).

Clinically, there have been a couple of studies using hCG in the treatment of GVHD: one in cGVHD (123) and another in aGVHD (124, 125). In the cGVHD treatment study, active cGVHD localized at the skin, subcutaneous tissue, joints, or gastrointestinal tract that was refractory or intolerant to glucocorticoid therapy improved substantially in 12 of 20 patients treated with hCG. In the aGVHD study at the University of Minnesota by Holtan et al., they selected urinary-derived hCG (uhCG; as opposed to recombinant hCG) due to the additional growth factors that could aid in tissue repair and inflammation resolution, not just immunosuppression. One of these is epidermal growth factor (EGF), which is essential in tissue repair and has receptors in the crypts (126). In some preclinical models of intestinal damage, supplemental EGF has been shown to enhance gut epithelial restoration after radiation (127). This is also important since there is evidence suggesting that damage to the gut by chemotherapy or radiation plays a role in the initiation and amplification of systemic disease in GVHD by increasing the translocation of inflammatory stimuli, which promotes the cycle of further inflammation and cytokine storm (128). Holtan et al. examined plasma samples from patients enrolled in the BMT CTN 0402 collected at pre-SCT baseline, day +28, and day +100 during the course of the study and found that pre-SCT low EGF was associated with a higher risk of developing grade II and IV aGVHD by day +100 and increased NRM (129). At the 63rd ASH Annual Meeting in 2021, Dr. Holtan presented the results of a phase II clinical trial (NCT02525029) adding uhCG/EGF to standard therapy in two cohorts of patients, new onset Minnesota high-risk aGVHD and aGVHD requiring second-line therapy, extending from the previously published phase I study (125). In the phase I study, dosing began at 500 units hCG/m^2^ (level 1) and then went up to 1,000 units hCG/m^2^ (level 2) and 2,000 units hCG/m^2^ (level 3) given subcutaneously every other day for 1 week in the high-risk, newly diagnosed aGVHD and 2 weeks in the second-line aGVHD. Patients with complete or partial response were allowed to continue maintenance dosing of uhCG twice weekly for 5 weeks. Treatment was tolerated except for adverse events expected in these patients with GVHD, and only edema was considered a treatment-related adverse event. In the phase II report (50), there were 44 patients in total, with 22 in the high-risk aGVHD group and 22 in the second-line aGVHD group, with 52% having stage III–IV lower GI and 75% grade III–IV aGVHD. Despite all these high-risk features, the response rate (complete/partial response) at day +28 was encouraging at 68% (57% complete and 11% partial). Among patients who died, the reason for death was aGVHD (n = 9), relapse (n = 9), infection (n = 3), and organ failure (n = 2). Only one dose-limiting toxicity occurred (one cerebral venous sinus thrombosis, resolved with anticoagulation). In 2020, the FDA granted Orphan Drug Designation to uhCG/EGF for the treatment of aGVHD. Taken together, these studies provide a rationale for adding uhCG/EGF to PTCy after SCT from MMUDs, so we have started our phase I study of PTCy and uhCG/EGF as GVHD prophylaxis for MMUD PTCy2HCG3 (NCT04886726).

### 3.6 Lithium

Intestinal epithelial cells are important physical and chemical barriers that maintain healthy gut and microbiota interactions. This occurs through sophisticated homeostasis mechanisms that involve many functionally different cell populations in the crypt including but not limited to Paneth cells, intestinal stem cells, telocytes, and others. Before reviewing lithium specifically, it is important to review glycogen synthase kinase-3 (GSK3), which is different from other usual kinases because it is inhibited rather than activated by a stimulus. It is also involved in Wnt signaling and recently attracted the interest of many investigators in different fields like Alzheimer’s disease and cardiovascular disease (130).

Beurel et al. (131) showed that GSK3 might be involved in the early differentiation of Th17, and Klamer et al. showed that using a small molecule inhibitor of GSK3, 6-bromoindirubin 3′-oxime, prevents lethal GVHD in a humanized xenograft model in mice (132). Investigators at Fred Hutchinson Cancer Center designed a pilot study to examine the double effect of lithium in Wnt signaling to help intestinal crypt proliferation and mucosal healing and the inhibition of GSK3 in inflammatory cells to modulate the inflammatory response (52). Study patients received extended-release lithium carbonate tablets starting at 450 mg orally per day. Initially, the dose was adjusted, up to three doses daily, to maintain the serum lithium trough concentration at 0.8 to 1.2 mmol/L. Subsequently, the dose was restricted to once or twice daily and adjusted to maintain serum concentrations of 0.5 to 1.0 mmol/L. They reported a complete response of 67% when lithium administration was started promptly within 3 days of endoscopic diagnosis of denuded mucosa. When lithium was started promptly and less than 7 days from salvage therapy for refractory GVHD, the complete response was 80%. Toxicities included fatigue, somnolence, confusion, or blunted affect in 50% of the patients. Since Wnt signaling is critically required for intestinal cell regeneration, in a murine model, Koyama et al. (133) examined the effect of giving lithium from day −2 to day +7 post-T-cell replete transplant and showed that lithium decreased GVHD in the ileum. However, little effect was observed in the liver or skin, so its effect is probably more via an effect on Paneth that subsequently promotes intestinal stem cells rather than an effect on alloreactive T cells.

## 4 Discussion and future directions

Benjamin Franklin famously advised fire-threatened Philadelphians in 1736 that “an ounce of prevention is worth a pound of cure”. Prevention of GVHD not only helps decrease health care expenditure but also improves the quality of life for SCT recipients. However, prevention of GVHD might not be one-size-fits-all. There are so many variables that should be taken into consideration when thinking of GVHD prevention, especially when more options will be available. For example, in high-risk malignancies in pediatrics with ages 18 and younger, with MAC BM PTCy, there was a higher relapse probability (49%) compared to children historically receiving CSA/MTX (26%, p = 0.03) (134), possibly since pediatric patients have the advantage of having an active thymus, which might help in decreasing the risk of GVHD and infections (135, 136). However, older patients have age-related comorbidities that put them at higher risk for infections and organ toxicity. These changes involve immunosenescence (137), which is the decline in immune function, and inflamm-aging (138), which is a state of chronic sterile inflammation, in addition to other changes related to microbiota, cytokine production, and decreased antigen presentation (139). Older patients can also have irreparable DNA damage within telomeres in non-proliferating, non-mitotic tissues like cardiomyocytes, neurons, and osteocytes (140), which might affect some side effects related to GVHD medications. GVHD prevention approaches should likely be adapted between non-malignant and malignant diseases. With many additional variables to consider, there is a growing need for tools like machine learning to help physicians predict outcomes and stratify patients for personalized transplant approaches.

In the treatment of GVHD, more knowledge about pathophysiologic mechanisms in immune and regenerative pathways, in addition to the microbiome and enteroendocrine systems of the gut, may help provide patients with innovative strategies in the first-line treatment of GVHD. This is especially important for high-risk patients who have historically received prolonged high-dose corticosteroids. This is a huge unmet medical need. Filling this gap will help decrease immunosuppression, improve responses, and increase quality of life.

**Figure 1:**
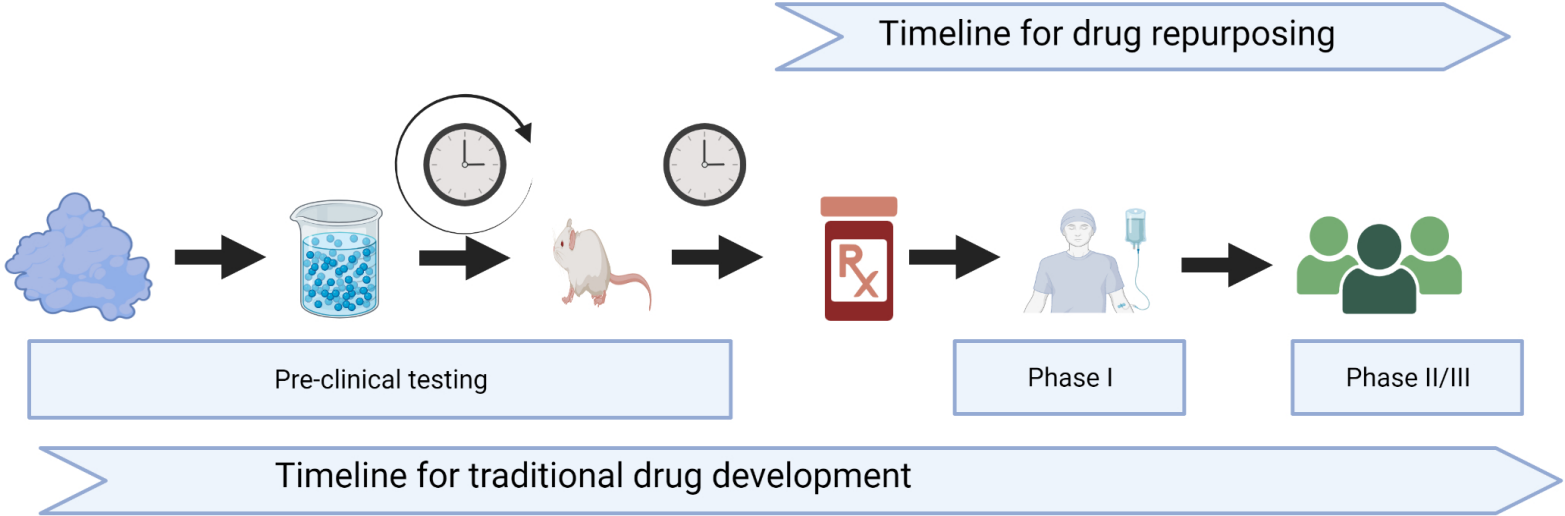
Timeline for traditional drug development versus drug repurposing. Created with BioRender.com.

The process of traditional drug development is a time-consuming and expensive process that includes 2–5 years of preclinical studies and toxicology, followed by a minimum of 3–5 years of phase I–III clinical trials, before entering the registration and marketing phases (**Figure 1**). In addition, there is a high risk of failure from the many drugs that start in the basic and discovery preclinical phases, and only one to two become eventually approved for indication. Repurposing old drugs with an established safety profile in GVHD prevention and treatment might help these patients with less expensive, more widely available options.

## Author contributions

SF and SH designed, wrote, and edited the manuscript. All authors contributed to the article and approved the submitted version.
